# Telerehabilitation for people with breast cancer through the COVID-19 pandemic in Chile

**DOI:** 10.3332/ecancer.2020.1085

**Published:** 2020-08-05

**Authors:** Williams Mella-Abarca, Valentina Barraza-Sánchez, Karol Ramírez-Parada

**Affiliations:** 1 Carrera de Kinesiología, Departamento de Ciencias de la Salud, Facultad de Medicina, Pontificia Universidad Católica de Chile, Santiago, 782-0436, Chile; 2 Physical Therapist, Casa Salvador Medical Center, Santiago, Chile; 3 Doctoral Program in Clinical Medicine and Public Health, University of Granada, Spain

**Keywords:** COVID-19, telerehabilitation, breast cancer

## Abstract

Adapting face-to-face physical therapy consultations in cancer care to a model of telerehabilitation has been necessary, given the imminent spread of the COVID-19 pandemic. In this respect, the current model of telerehabilitation for people with breast cancer can be described as a method of continuing physical therapy treatment, in a public hospital with limited resources.

## Introduction

Coronavirus 2019 (COVID-19) was declared a worldwide pandemic by the World Health Organization (WHO) on 11 March [1], and to date (June 2020) there are around 9.5 million confirmed cases in the world [2]. As of June 2020, this disease, caused by the SARS-CoV-2 virus, has been responsible for around 480,000 global deaths [2].

In Chile, the first reported case of COVID-19 was on 3 March, 2020. By 25 June, there were more than 250,000 confirmed cases [3]. The virus has so far been concentrated in the metropolitan area, accounting for more than 80% of confirmed cases [3]. Full lockdown of the metropolitan area was implemented on 15 May and was in force until June 26, with the possibility of a further 7 days of extension.

The pandemic has required a change in the day-to-day functioning of healthcare systems, both in Chile and throughout the world. The number of face-to-face outpatient appointments has been reduced due to the growing spread of the virus, which affects people with chronic illnesses, such as hypertension, diabetes and cancer, among others. In the case of people with cancer, medical and rehabilitative care has been changed, from suspending their treatment to changes in care protocols [4, 5].

The aim of this article is to describe a model of physical therapy using telerehabilitation for people with breast cancer (BC) during the COVID-19 pandemic in Chile.

### People with cancer during the COVID-19 pandemic

It has been mentioned that people with cancer are at high risk during the COVID-19 pandemic [4–7]. However, it seems that the risk of infection and complications associated with the virus apply only to immunosuppressed persons—this means those undergoing active treatment or people with certain types of tumours (haematologic and lung cancers). Moreover, emerging research indicates that hypertension and diabetes are conditions that increase the risk of infections and complications [7].

### Models of telerehabilitation for people with chronic diseases

Telehealth, telemedicine and telerehabilitation are not new concepts. There are reports which indicate that telerehabilitation may have begun in the 1950s [8], with the popularity of telehealth as it is known today beginning to grow in the 1970s [9]. In this paper, we will use the concepts outlined in the practical guide to telerehabilitation by the Chilean Physical Therapy Board (Colegio de Kinesiólogos de Chile) [10]. In other words, telehealth as a global concept, telemedicine as a medical occupation and telerehabilitation as being within the remit of a physical therapist. The different models of telehealth can be found in Table 1*.*

### Telerehabilitation for people with breast cancer

The research on models of telerehabilitation for people with BC is scarce. In general, the available evidence is positive regarding early detection of lymphedema, improving the quality of life, reducing pain and fatigue and improving muscular strength in survivors of BC [11, 12]. It also increases adherence, motivation and appears to be a cost-effective intervention [13, 14]. Recent reports outline that the current health crisis presents an opportunity to progress towards telerehabilitation [11–13].

## A model of physical therapy for people with breast cancer

The Complejo Asistencial Dr Sótero del Rio is the public referral hospital for the entire southeast side of the metropolitan area. It primarily accommodates the population in the lower 50% by socioeconomic classification (the population with the lowest income and highest vulnerability). It benefits from a Breast Pathology Unit, where every patient over the age of 18 and diagnosed with breast cancer receives breast and axillary surgery, chemotherapy, radiotherapy and hormone and biological therapy [15–17]. All medical treatment, including physical therapy, is free for patients, as it appears on the list of specific allowances in the Explicit Health Guarantees for breast cancer [18].

Since 2015, we implemented an early and prospective physical therapy model. This model focuses on decreasing the side effects of breast cancer surgery, which can include lymphedema, axillary web syndrome, limited movement in the upper limbs and reduced muscle strength [17]. However, given the health risk, face-to-face out-patient appointments have been restricted, and telerehabilitation has been adopted.

### Adapting the model of physical therapy for people with breast cancer to telerehabilitation

**Figure 1.**
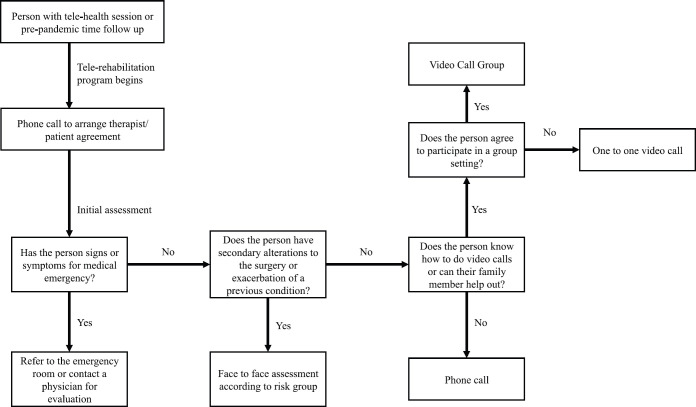
Initial assessment before beginning the program via telerehabilitation.

**Figure 2.**
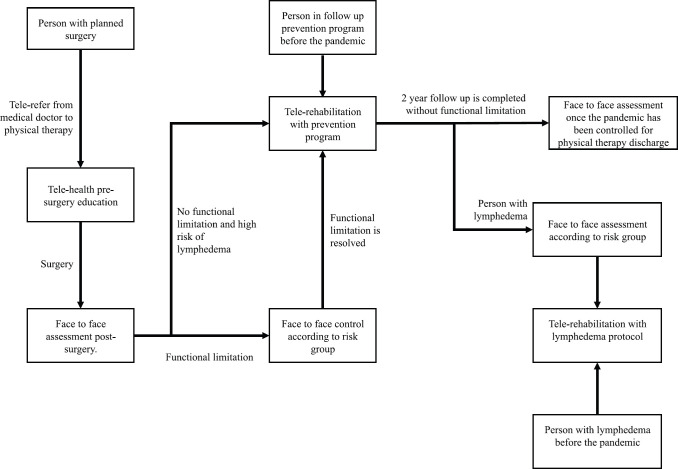
Follow-up care for new patients and those with prior examinations. (*) corresponds to periods in which it is possible to investigate a medical emergency, for which reason referral to a doctor must occur.

The model of telerehabilitation for people with breast cancer in the Complejo Asistencial Dr Sótero del Río has been in place since April 2020, as a hybrid- or mixed-model type (Table 1) and, as with face-to-face sessions, is free of charge for the patients. To join the telerehabilitation program, an initial assessment is required, as shown in Figure 1. Medical emergencies, exacerbation or functional disorders are evaluated, and the arrangements for future remote consultations (therapist – patient agreement) are established. This first meeting is carried out by telephone and lasts approximately 20 minutes. Following the patient’s agreement to future checks and receipt of their verbal consent, the next steps are determined according to individual needs (Figure 2). This may be a phone call, an individual video call via a mobile device (computer or smartphone), or a group video call, depending on the availability and convenience of the individual’s devices. Follow-up checks last on average 50 minutes. The frequency of these checks will depend on the risk posed by visiting the health centre (Table 2).

People who have undergone axillary dissection surgery or who have a limited range of movement in their upper limbs (resulting from axillary web syndrome or otherwise) should attend a face-to-face check in addition to asynchronous education, whereby a functional mobility assessment tool is used on the patient’s shoulder and their physical therapy treatment is started. This will then be supervised remotely every 15 days.

### Telemonitoring following the model of physical therapy for people with breast cancer

All checks are carried out synchronously with the patient, though information is submitted via a web page without the therapists’ direct supervision (link: www.oncoactivate.cl). This information is communicated asynchronously during the program [19]. The transition from the face-to-face model to telerehabilitation has been progressive and in line with the increasing number of infections in the country.

### Pre-surgical assessment for breast cancer

People recommended for breast cancer surgery are referred for physical therapy, with teleconsultation from the Breast Pathology Unit. A synchronous consultation is scheduled with the patient for pre-operative assessment and education. During the assessment, functional mobility of the patient’s shoulder is observed and recommendations regarding physical functionality will be given for the pre- and post-operative period. The patient is given a therapeutic exercise plan with particular emphasis on the upper limbs and receives counselling on the benefits of physical activity and general self-care.

### Prevention of lymphedema

People on the lymphedema prevention program are monitored remotely every 3 months. On the initial evaluation, a verbal questionnaire is conducted regarding signs and symptoms associated with secondary alterations to the medical treatment, with particular emphasis on screening for lymphedema (heavy feeling in the arm ipsilateral to the surgery, increase in arm volume, skin quality, etc.). They are given, and analyse, a therapeutic exercise plan with particular emphasis on exercises that favour venous and lymphatic return from the upper limbs, and receive counselling on the benefits of physical activity and general self-care. Also, the patient is educated about how to measure the girth of upper limbs to obtain their volumes. Obtaining the volume is carried out by measuring the perimeters of the arm, measured using a tape measure at certain anatomical points. We suggest using the Cleveland Clinic webpage: *Breast Cancer – Arm Volume Calculator* which advises using six anatomical points [20]. This webpage is free-to-use and, using a mathematical calculation, it gives the difference in volume between the arms. If the person has more than 10% or 200 ml difference in volume between the affected arm and the other arm, the condition lymphedema is considered as present. The patient is recommended to carry out asynchronous self-assessments once a month without the supervision of the therapist and inform them of any changes.

### Lymphedema

People with lymphedema are examined once a month with the aim of keeping their lymphedema stable. The sessions consist of assessing the lymphedema (presence of fibrosis, changes in volume and subjective sensation of symptoms: heaviness, skin colour and consistency, among others), patients are educated and/or supervised on manual lymphatic self-drainage, therapeutic exercise is supervised to promote lymphatic and venous return of the upper limbs. Instruction is given on how to measure the girth of upper limbs to obtain the volumes with the same protocol as in the prevention of lymphedema. Also, advice is given on the correct use of compression treatments and skin care. Likewise, with all follow-up care, advice is given on the promotion of physical activity and general self-care.

### Implementation of the model

COVID-19 arrived in Chile in March 2020, nevertheless, our tele-rehabilitation model began to be rolled out widely in April of the same year. The Dr. Sótero del Río Health Care Complex uses a digital platform which permits remote access to the patient’s clinical file. If we consider the care delivered since April until 22 June, 226 care events have been recorded of which 63% correspond to tele-rehabilitation. Of this care, 8 instances correspond to pre-operative checks (7 via tele-rehabilitation, that is to say 88%), 26 to follow-up appointments for Axillary Web Syndrome (5 by tele-rehabilitation, 19%) 72 for people with lymphedema (51 by tele-rehabilitation, 71%), 107 for prevention of lymphedema (74 by tele-rehabilitation, 69%) and 13 for other consultation reasons such as painful conditions, assessments etc (5 by tele-rehabilitation, 38%). The demographic characteristics of the 118 people that have received tele-rehabilitation can be found in Table 3. Regarding the number of instances of care provision for these people, 95 (80%) only received it on 1 occasion, 22 (19%) on 2 instances and 1 person (1%) on 3 instances.

The tele-rehabilitation program at the Dr. Sótero del Río Health Care Complex has had a high level of acceptance and satisfaction, both by the patients and the physiotherapists, since during pandemic face-to-face checks involve an infection risk both for the service user and the health professional. Patients value the continuation of their sessions and having therapists willing to help and resolve doubts in case of needing to investigate any change in their condition.

### Enablers and blockers

We can pick out six variables which present as facilitating factors to implement this model of tele-rehabilitation: 1) Having an electronic medical record system which allows legal record-keeping and to establish concrete rehabilitation objectives. 2) To have a multidisciplinary team which maintains good communication. 3) The growth in use of technology in the country (wide internet access and smartphones). 4) Obligatory quarantine, in many cases it enables the company of a family member to assist the patient to manage the technology. 5) Creation of a webpage with facilitates asynchronous contact with the person. 6) High levels of commitment and motivation of therapists/patients to participate in this new model.

The barriers to implementing the telerehabilitation model relate to the difficulty in coordinating the synchronous therapist – patient check-ups, and the inability to perform a complete physical examination, which for physiotherapists is essential, especially as palpation is prevented, for which they now rely on the patient’s perception.

### Ethical implications and safe practice

Everyone who participated in the telerehabilitation program has done so of their own free will and fully aware of their decision. The benefits of having therapy from their houses and the risks involved in attending face-to-face checks, given the health crisis, were explained to them. There is a moral obligation to protect lives, both one’s own and of others, therefore, only in those cases where not assisting in person would involve a greater risk than the risk of infection from COVID-19 were they offered face-to-face therapy, taking every possible protective measure both for the patient and the healthcare staff.

## Conclusion

Due to the current COVID-19 pandemic, many people with chronic conditions have seen their face-to-face health checks changed or suspended to reduce the risk of infection from the virus. However, in many cases, this means a deterioration in the person’s condition, which could be reduced by introducing telehealth as a tool in this time of crisis. Specifically, in breast cancer, the early intervention and forward-facing models of physical therapy have proven to be effective in the reduction of post-operative breast complications, and the experience in a Chilean hospital shows that it is possible to implement this model in the case of telerehabilitation.

## Conflicts of interest

The authors declare that there is no conflict of interest.

## Funding

No funding was received for this paper.

## Figures and Tables

**Table 1.** Models of Telerehabilitation in chronic conditions.

Time lapses	
Synchronous	Consultation between the professional and the patient carried out in real time. Consultation carried out from afar, but at the same time.
Asynchronous	The consultation is not carried out in real time, that is to say, the professional and the patient do not meet at the same time.
Hybrid or mixed	Combination of synchronous and asynchronous consultations.
**Types of care**	
Teleconsultation	Classic consultation between the professional and patient. Teleconsultation has the feature of being carried out from afar utilising available technology.
Remote interdisciplinary consultation	Referral between different types of professional via an electronic system, subsequently the patient receives a teleconsultation from another specialist.
Telemonitoring	Monitoring the patient through a device. In this type of care information is transmitted direct to the professional.

**Table 2.** Classification of women with breast cancer according to their risk of infection from COVID-19.

Risk	Clinical-Demographic Criteria	Face-to-face follow-up
Low	- People younger than 40 without comorbidities	Onsite consultation at any time, with bio-security measures
Medium	- People aged 41 to 59 without comorbidities who are having active hormonal, immunological or radiotherapy treatment	Face-to-face consultation in case of high or moderate priority. Emphasis must be placed on biosecurity measures
High	- People aged 60 years or over with or without comorbidities - People undergoing active chemotherapy and/or corticosteroid treatment - People with at least 1 relevant comorbidity, independent of age	Avoid face-to-face consultations. Carry out all care remotely except in case of high priority, which must be referred immediately to the emergency department or the treating medic.

**Table 3.** Demographic characteristics of the 118 people who participated in tele-rehabilitation.

Characteristics	Data n (%)
Age 30 or under 31 to 40 years 42 to 50 years 51 to 60 years 61 or over	6 (5) 11 (9) 21 (18) 28 (24) 52 (44)
Period of follow-up care 6 months or less 6 months to 1 year 1 to 2 years 2 years or more	31 (26) 27 (23) 37 (32) 23 (19)
Type of breast surgery Total mastectomy Partial mastectomy Awaiting procedure	50 (42) 62 (53) 6 (5)
Type of axillary surgery Sentinel node biopsy Axillary dissection Not yet	15 (13) 97 (82) 6 (5)
Chemotherapy Neoadjuvant Adjuvant No Awaiting committee decision	48 (41) 39 (33) 23 (19) 8 (7)
Radiotherapy Yes No	83 (71) 34 (29)
Hormone therapy Yes No	71 (63) 44 (37)
Immunotherapy Yes No	25 (21) 92 (79)
